# Does Cyberostracism Reduce Prosocial Behaviors? The Protective Role of Psychological Resilience

**DOI:** 10.3390/ijerph19074388

**Published:** 2022-04-06

**Authors:** Linyu Shi, Hao Li, Lianqiong Huang, Yubo Hou, Lili Song

**Affiliations:** 1 School of Psychological and Cognitive Sciences, Beijing Key Laboratory of Behavior and Mental Health, Peking University, Beijing 100871, China; ant123@163.com (L.S.); h_lianqiong1992@163.com (L.H.); 2 Plateau Brain Science Research Center, Tibet University, Lhasa 850000, China; futanghu888@126.com; 3 Institute of Oxygen Supply, Tibet University, Lhasa 850000, China; 4 Institute of education, Tibet University, Lhasa 850000, China; 5 School of psychology, South China Normal University, Guangzhou 510000, China; 6 CAS Key Laboratory of Mental Health, Institute of Psychology, Beijing 100101, China; 7 Department of Psychology, University of Chinese Academy of Sciences, Beijing 100049, China

**Keywords:** cyberostracism, prosocial behavior, psychological resilience, online self-help intervention, temporal need-threat model

## Abstract

To reduce the negative consequences of cyberostracism on prosocial behaviors, we developed a coping strategy based on psychological resilience, and revealed its effectiveness in combating the adverse effects of cyberostracism on prosocial behavior through two studies. Study 1 demonstrated that psychological resilience could mitigate the negative impact of cyberostracism on prosocial behaviors through experimental manipulation. By targeting continuously ostracized people with low resilience for an online self-help resilience intervention program, Study 2 confirmed that psychological resilience was effective in alleviating the detrimental effects of cyberostracism. These studies not only help us to recognize the negative effects of cyberostracism, but also extend Williams’ temporal need–threat model of ostracism in the context of online ostracism. As emerging technologies represent a promising new approach to intervention delivery, the most valuable contribution of this study is that we developed an online self-help psychological resilience intervention program that showed encouraging therapeutic effects and advantages for assisting in caring for a larger population of people who are at elevated risk for being cyberostracized.

## 1. Introduction

Currently, online social media such as Facebook, Tiktok, Twitter, Weibo, and WeChat are increasingly penetrating our lives; although these media facilitate interpersonal interactions, they also increase the risk of problematic internet behaviors, such as internet addiction, cyberbullying, or cyberostracism.

Cyberostracism is a form of cyberbullying [1]. The characteristics of the online environment might promote moral disengagement [2,3,4], and reduce social–emotional cues, thus leading to more intentional or unintentional network transgressions [5,6]. Specifically, cyberostracism is the phenomenon and process in which individuals’ needs for belonging and relationships are hindered through the disruption of social connections during online social interactions [7]. Although being generally considered a form of cyberbullying, cyberostracism more emphasizes any intended or perceived ostracism in electronic-based interactions other than face-to-face [8], which might not be direct aggression or intentional targeting behaviors.

Although cyberostracism is the manifestation of ostracism on the internet, the characteristics of online social interactions, such as asynchrony, strong technological dependence, anonymity, and the absence of social clues, also make cyberostracism more illusory and uncertain than ostracism in real life [9,10,11]. Therefore, the deleterious impact of cyberostracism on individuals’ psychosocial adaptation is more severe and long-lasting [12]. In addition to the negatively affected psychosocial adaptation and emotional state of ostracized individuals [12,13,14], a growing body of evidence shows that cyberostracism makes ostracized individuals have more maladaptive behaviors, such as aggression [15,16], and fewer prosocial behaviors [17]. Numerous studies have proven that cyberostracism damages the victims’ mental and emotional health [12,14]. However, more noteworthy is that increased aggression or decreased prosocial behaviors caused by cyberostracism could further cause victims to become perpetrators. Therefore, how to prevent this dangerous process is the goal of this study.

### 1.1. Cyberostracism and Prosocial Behavior

Prosocial behaviors, also known as altruistic behaviors, refer to behaviors that meet social expectations, and have no obvious benefits to the actors, but instead involve actors voluntarily offering benefits to the recipients of the behaviors [18]. In previous research, there have been discrepancies in ostracized individuals’ behavioral responses. According to the temporal need-threat model of ostracism [19,20], after being ostracized, ostracized individuals experience three stages: (1) reflexive stage (immediate), (2) reflective stage (coping), and (3) resignation (long-term). In the reflexive stage, ostracism impairs the ostracized individual’s four fundamental psychological needs, i.e., belonging, self-esteem, control, and meaningful existence. In the reflective stage, ostracized individuals make a series of efforts to escape the negative influence of ostracism according to the type of thwarted needs. Ostracized individuals with thwarted power needs (control and meaningful existence) are more likely to behave antisocially, whereas those with thwarted relationship needs (belonging and self-esteem) are prone to behave prosocially.

However, some researchers have also explained the behavioral responses of ostracized individuals from other perspectives. On the one hand, some researchers believe that ostracism interferes with ostracized individuals’ emotional responses, and impairs their empathy for others, thus leading to the destruction of their prosocial behaviors [21,22]. From a cognitive perspective, Buckley and colleagues [23] demonstrated that ostracism affects ostracized individuals’ cognitive evaluation of themselves and others, i.e., there is a significant reduction in their self-esteem, and an increasing negative evaluation of the perpetrator, the latter of which usually leads to biased attitudes and behaviors [24], thus causing a decrease in prosocial behaviors. In terms of self-regulation, Baumeister et al. [25] found that ostracism impaired individuals’ self-regulation; compared with the included group, the ostracized group showed more out-of-control behaviors, such as excessively consuming unhealthy drinks and snacks, more easily becoming discouraged, more quickly giving up on difficult tasks, and having a harder time resisting interfering information. A two-year longitudinal study also demonstrated that ostracism impeded the development of children’s self-regulation in the long run, which, in turn, increased their susceptibility to being ostracized [26]. It has even been shown that simply observing others being ostracized can also damage an individual’s self-regulation [27]. On the other hand, some researchers hold the opinion that ostracism improves individual perspective-taking [28] and the theory of mind [29], prompting them to shift from being egocentric to other-centric, and thus, behave prosocially [30]. However, regarding cyberostracism, it is unclear whether ostracized individuals increase or decrease prosocial behaviors. The present study assumes that cyberostracism may be primarily associated with the characteristics of online social interactions.

It is undeniable that individuals ostracized online suffer psychological feelings similar to those ostracized offline. Studies have shown that, similar to offline ostracism, cyberostracism also threatens individuals’ basic psychological needs, and induces negative emotions [31,32,33,34]. In addition, similar to real-life ostracism, cyberostracism has been proven to elicit “social pain” in victims [35], which usually distracts victims’ attention [36], making them more likely to ignore others’ needs and thereby reduce their prosocial behaviors. According to the above findings, it can be seen that the temporal need–threat model of ostracism is also applicable to cyberostracism.

However, unlike face-to-face ostracism, online social communications have distinctive new features, such as the mutual invisibility of the interacting parties, anonymity, and lack of personalized information and social contextual clues [7,37] These new features of online social interactions suggest that there may be differences between cyberostracism and real-life ostracism. First, as ostracism has a tremendous negative impact on individuals’ self-regulation, in a depersonalized network situation, cyberostracism even weakens ostracized individuals’ ability for behavioral regulation. Individuals suffering from cyberostracism do not have to worry about real-life social situations such as offline ostracism [37], which makes it easy for them to deviate from moral constraints in action. Therefore, they are more prone to feel anger after being ostracized, and more likely to express anger without hesitation [35,38]. Such out-of-control behavior caused by the decline in self-regulation may be manifested on the internet with more aggressive [15,16,39] and less prosocial [40] behaviors. Second, existing evidence has also illustrated that the bluffing effect is more likely to occur in cyberostracism than in offline ostracism; that is, ostracized individuals attempt to maintain their participation in group discussions in the online chatting room by provoking other group members, to avoid being ostracized from the chat and to increase their sense of control [9,11]. This kind of response in adopting more extreme strategies to gain attention and recover thwarted needs may be precisely caused by these distinct characteristics of online interaction that make the ostracized behave in a more unconstrained way.

In addition, the physical isolation brought by the internet also causes ostracized individuals to show cognitive disintegration and the dehumanization effect [41]. That is, ostracized individuals’ ability to regulate behaviors is weakened, and further, makes them deny the fact that others have human common characteristics, such as emotional richness, enthusiasm, and cognitive flexibility [41]. They believe that others are dull, indifferent, and rigid, which may lead to a decrease in prosocial behaviors [17]. Previous research has shown that the anonymity and deindividuation of online interactions usually lead to increased group ostracism and the more serious distress of stigmatized group members after ostracism [8].

Moreover, successful interaction depends on technology [42]; thus, poor communication between individuals due to network technical failures also greatly increases the frequency of “illusionary ostracism”, which causes individuals to be vulnerable to experiencing uncertainty [43], and to engage in introspection that is harmful to themselves, thus negatively impacting their mental states and behaviors [11], and further reducing prosocial tendencies [43]. Therefore, we propose the following hypothesis:

**H1.**  Cyberostracism will reduce prosocial behaviors.

### 1.2. Protective Role of Psychological Resilience

Kaplan et al. [44] argued that risk factors do not necessarily cause individuals to experience severe physical or mental symptoms, and that what matters is whether the individual has protective factors to deal with risks. An ostracized individual’s behavioral responses may also be related to protective factors, such as his or her characteristics. The existing literature has shown that compared with individuals with low levels of self-esteem, individuals with high levels of self-esteem suffer less from the negative effects of ostracism [45], and their prosocial behaviors are not significantly decreased [46]. Similar to self-esteem [47], psychological resilience is also a positive psychological trait. It refers to the positive development and adaptive representation of complex and dynamic interactions among risk factors, resource factors, and psychosocial functions over time when an individual has experienced or is experiencing severe stress and adversity [48]. A variety of studies have shown that psychological resilience has a positive impact on reducing major diseases, adversity, and stress disorders [49,50,51]. Recent research has shown that psychological resilience relieves the detrimental influence of cyberbullying on victims’ mental health [52]. Therefore, we expect that psychological resilience alleviates the harmful impact of cyberostracism on prosocial behaviors.

First, life events are common psychosocial stressors that affect individuals’ physical and mental health [53]. Cyberostracism can also be seen as a negative life event and a risk factor, which generates negative effects (e.g., social pain and negative emotions) for ostracized individuals. However, resilience helps relieve these negative influences [50,54]. A high level of psychological resilience helps ostracized individuals mobilize various protective elements to coordinately respond to stressful events [55,56], thus making them successfully cope with adversity, major diseases, and stress disorders such as cyberostracism [50,51]. For example, it has been found that resilience reduces the unfavorable effects of ostracism on depression [57].

Second, according to frustration–attack theory [58], if individuals who suffer from cyberostracism have no appropriate coping styles, they may engage in aggressive behaviors to deal with adversity under the guidance of attack clues. However, a high level of psychological resilience enables individuals to consciously accept the frustrating event, i.e., cyberostracism, and proactively employ effective problem-solving tactics to cope with the setback [59].

Additionally, according to the temporal need–threat model of ostracism [20], an individual’s behavioral responses after cyberostracism may also depend on what needs are threatened. In general, if relationship needs are threatened, ostracized individuals will compensate for these needs by behaving prosocially. Conversely, if competence needs are thwarted, they will behave antisocially to consolidate the thwarted needs. Psychological resilience prevents individuals from experiencing risk factors such as relationship threats by reshaping relationships and increasing social support when encountering adversity [60,61]. Furthermore, psychological resilience also helps mobilize individuals’ protective factors (such as self-esteem and sense of control) to confront negative stimuli [55,56]. These findings remind us that psychological resilience may be conducive to adjusting ostracized individuals’ relationship needs to cope with cyberostracism, making them feel less threatened by competence needs, thereby reducing antisocial behaviors, and even increasing prosocial behaviors. Therefore, we propose the following hypothesis:

**H2.**  Resilience mitigates the negative effects of cyberostracism on prosocial behaviors.

### 1.3. The Present Research

The present research investigated the relationship between cyberostracism and prosocial behaviors, and the moderating role of psychological resilience using two studies. Specifically, Study 1 examined the causal relationship between cyberostracism and prosocial behaviors, as well as the moderating role of resilience, through experimental manipulation. To verify the protective role of resilience, Study 2 conducted a psychological resilience intervention among people who experienced long-term cyberostracism and had low levels of resilience. We hypothesized that cyberostracism would reduce prosocial behaviors, and that resilience would moderate these negative effects.

## 2. Study 1

Study 1 had two goals. First, we aimed to examine the hypothesized associations between cyberostracism and prosocial behavior by manipulating cyberostracism. Second, we investigated the moderating role of psychological resilience between cyberostracism and prosocial behavior.

### 2.1. Method

**Participants**. First, 1280 participants were recruited online via Credamo to complete a questionnaire including the Life Event Scale (*LES*), Life Satisfaction Scale (*LSS*), Positive and Negative Affect Schedule (*PNAS*), and Connor-Davidson Resilience Scale (*CD-RISC)* (see the following part for details of these measures). A total of 99 participants failed the quality check questions and were thus excluded, which resulted in 1181 participants. Second, we conducted screening and grouping processes according to two vital standards of resilience, i.e., the severity of stress/adversity that they faced, and the positive outcome of psychosocial adaptation that they developed [62,63,64,65,66,67]. Specifically, 696 participants were selected based on their scores on the *LES* being higher than the average, and then they were divided into a high-psychological resilience group (H-group, *M* = 42.66, *SD* = 3.02, *N* = 150) and a low-psychological resilience group (L-group, *M* =30.72, *SD* = 5.08, *N* = 150), according to their psychosocial adaptation scores (*LSS + PNAS*) being higher or lower than the average score with a standard deviation. We compared their scores on the *CD-RISC*, and found that participants reported higher psychological resilience in the H-group (*M* = 42.66, *SD* = 3.02) than in the L-group (*M* = 30.72, *SD* = 5.08), indicating that the screening and grouping of resilient participants was successful. Consequently, a total of 300 participants (*female* = 184, *M*_age_ = 27.99, *SD*_age_ = 5.08) continued to complete the follow-up procedures.

### 2.2. Materials and Procedure

**Life Event Scale**. The Stressful Life Event Scale (LES) developed by Yang and Zhang [68] was used to assess an individual’s stressful adversity. The scale contains 48 common life events in China, and covers risk factors for family life (28 items), work/study (13 items), and social and other aspects (7 items), and 2 blank items where participants fill in their experiences that are not listed in the scale. Participants were asked to judge whether the events they experienced in the list were good or bad for them, and to what extent these events affected them. The scale was scored on a 5-point scale. If the event was considered to be good and the impact was positive, then 0 was selected as reflecting not occurring or no negative impact. If the event was considered to be bad and the impact was negative, then 1–4 was selected (1 = mild negative impact, 4 = extreme negative impact). A higher total score reflects the greater mental stress that an individual is under, and the higher the stress adversity index. Sample items included “Spouse death,” “Dissatisfied with the current job,” and “Involved in civil legal disputes” (*Cronbach’s α* = 0.89).

**Psychological function.** Subjective well-being, as an indicator of a stable reflection of an individual’s good life and emotional state, is more consistent with the connotation of "sustainability" in psychological resilience [69]. In this study, the *LSS* and *PNAS* were used to evaluate the adaptive development status. The *LSS* was administered using 5 items adapted from Diener et al. [70]. The participants rated their level of agreement with statements (1 = strongly disagree to 5 = strongly agree). A sample item was “I am satisfied with my life”. The *PNAS* was administered using 20 items adapted from Watson et al. [71]. The participants rated the items on a scale from 1 = very slight or none to 5 = very strong. Sample items included “Interested” and “Irritable”. The Cronbach’s α coefficients of the two scales were 0.93 and 0.94. We computed the sum of scores of these two scales as subjective well-being (*Cronbach’s α* = 0.96).

**Connor-Davidson Resilience Scale (CD-RISC)**. To validate the screening and group process of resilience, participants were also asked to evaluate their psychological resilience using the *CD-RISC-10* Scale [72]. This scale has ten items, and scores on a 5-point scale, with 1 for “never” and 5 for “always” (*Cronbach’s α* = 0.93). A sample item was “Tend to bounce back after illness or hardship”.

**Manipulation of cyberostracism**. The participants were randomly assigned to conditions of cyberostracism and cyberacceptance. Under both conditions, the participants were guided to read the material in which the organizers of a New Year’s party issued an invitation online and summarized the event after the party (see Appendix B). In the cyberostracism condition, we instructed the participants to imagine that they were the organizer and that they had been ignored and rejected by users, whereas in the cyberacceptance condition, we asked the participants to imagine that they were the organizer and that they received much attention, support, and praise from users. This manipulation was used by Vandevelde and Mivahara [73], Pfundmair et al. [74], and Bernstein and Claypool [75]. To enhance the manipulation effect, we asked the participants to write down the emotional experiences they had just imagined in both conditions. As a manipulation check, participants indicated their agreement (1 = strongly disagree; 5 = strongly agree) on one item, “I feel ostracism in the above condition”.

**Prosocial behavior**. Five self-developed conditions about donation (see Appendix C) were used following Yao [76]. After reading each condition, we asked the participants two questions in turn: “Faced with this situation, if you only have 100 yuan, are you willing to help?” (1 = very unwilling to 5 = very willing); “How much are you willing to donate?” (1 = 0 yuan, 2 = 1–20 yuan, 3 = 21–40 yuan, 4 = 41–60 yuan, 5 = 61–80 yuan, 6 = 81–100 yuan). The order was counterbalanced across the five conditions. The Cronbach’s α coefficient for ten items was 0.93.

### 2.3. Results

First, we conducted a manipulation check of cyberostracism. The results showed that participants reported higher ostracism in the cyberostracism condition (*M* = 9.14, *SD* = 0.87) than in the cyberacceptance condition (*M* = 2.38, *SD* = 0.91)*,* *t* (298) = 65.65, *p* < 0.001, indicating that the manipulation of cyberostracism was successful.

**Figure 1:**
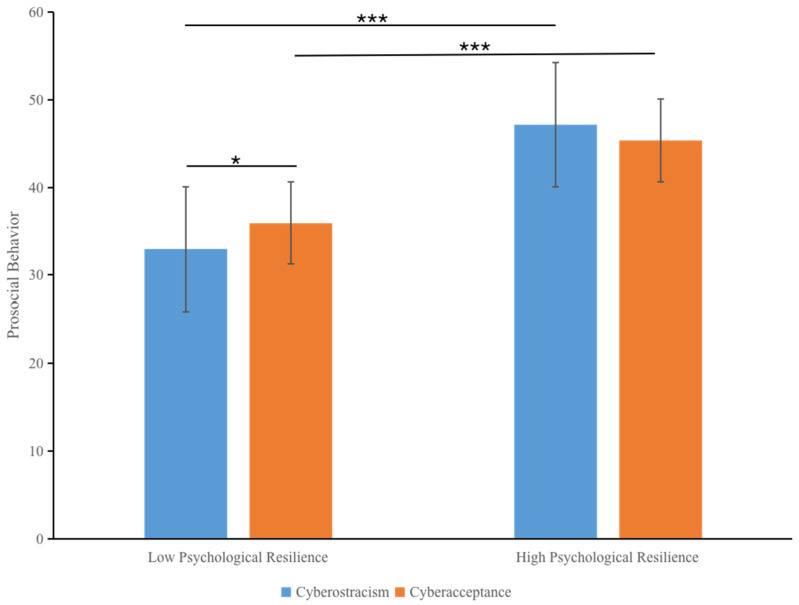
Effects of psychological resilience and cyberostracism on prosocial behavior. Error bars represent the 95% confidence interval. ***Note.*** * *p* < 0.05, *** *p* < 0.001.

A 2 (psychological resilience; between-participant) × 2 (cyberostracism; between-participant) mixed *ANOVA* on prosocial behavior showed significant main effects of psychological resilience (*F*(1, 289) = 92.17, *p* < 0.001, *p-η^2^* = 0.242). However, the main effect of cyberostracism (*F*(1, 289) = 0.46, *p* = 0.498, *p-η^2^* = 0.002) was not significant. The effect of the interaction between psychological resilience and cyberostracism was significant (*F*(1, 289) = 6.99, *p* = 0.009, *p-η^2^* = 0.024). As seen in Figure 1, a simple test showed that in the L-group, participants primed with cyberostracism (*M* = 32.92, *SD* = 9.08) scored lower on prosocial behavior than those primed with cyberacceptance (*M* = 35.93, *SD* = 7.48), *F*(1, 289) = 5.47, *p* = 0.020, *p-η^2^* = 0.019). However, in the H-group, participants showed no significant difference regardless of whether they were in the ostracism condition (*M* = 47.11, *SD* = 6.39) or acceptance condition (*M* = 45.36, *SD* = 6.60), *F*(1, 289) = 1.94, *p* = 0.165, *p-η^2^* = 0.007). From another perspective, participants in the H-group (*M* = 47.11, *SD* = 6.39) scored higher on prosocial behavior than those in the L-group (*M* = 32.92, *SD* = 9.08), *F*(1, 289) = 82.04, *p* < 0.001, *p-η^2^* = 0.221) in the cyberostracism condition. In addition, participants in the H-group (*M* = 45.36, *SD* = 6.60) also scored higher on prosocial behavior than those in the L-group (*M* = 35.93, *SD* = 7.48), *F*(1, 289) = 32.52, *p* < 0.001, *p-η^2^* = 0.101) in the cyberacceptance condition. 

## 3. Study 2

Study 1 found that cyberostracism reduced prosocial behaviors among individuals with low psychological resilience rather than those with high psychological resilience, indicating that psychological resilience can attenuate the potential negative effects of cyberostracism on prosocial behavior. One important follow-up question was whether the negative effect of cyberostracism on prosocial behavior could be alleviated by training the participants’ psychological resilience.

### 3.1. Method

**Participants**. A total of 601 participants recruited from Credamo completed the *Cyberostracism Experience* Scale, the *Depression–Anxiety–Stress* Scale, and the *Connor-Davidson Resilience* Scale (CD-RISC) (see subsequent sections for details on the scale information). Among them, we first selected 245 participants whose scores in cyberostracism were higher than the average. Next, we chose 187 out of the 245 participants who had a Depression–Anxiety–Stress score in the top 75%. Finally, 128 participants with a psychological resilience score in the top 70% from low to high were selected from the 187 participants. These 128 participants who scored high on Cyberostracism experience and mental disorder, but low in resilience, were randomly assigned to the intervention (*n* = 64) or control (*n* = 64) group. During the 21-day study, 63 valid participants (*female* = 32, *M*_age_ = 27.13, *SD*_age_ = 6.54) remained after removing the participants who were naturally lost, failed to pass the screening questions for seriousness, and had sloppy answers. Among these 63 participants, 32 were in the intervention group, and 31 were in the control group. 

**Materials and procedure**. This study was approved by the ethics commission of Peking University (#2015-03-03c). The participants were randomly assigned to the intervention or control group. In the intervention group, a self-developed 21-day online psychological resilience intervention program was used. Participants were required to complete 30-min training tasks related to psychological resilience every day. These tasks include two parts: one was predesigned psychological resilience reading modules, and the other was a structured diary writing assignment based on the above reading materials. In this process, participants were asked to record the current or recent events reflectively according to our tips, and elaborate or describe them in the way we required (see Appendix D). For example, on day 5, participants were asked to read a frustration file on several celebrities (such as Beethoven and Gorky) and some stories about optimism/pessimism, aiming to help them cultivate a dialectical view and positive attitude toward misery and setback. Furthermore, the post-traumatic growth was also delivered through several traditional Chinese documents, such as "The sharpened sword is from honed out; the plum blossom fragrance comes from the bitter cold" to motivate the participants to develop faith in the positive outcome of adversity. Then, they were asked to recall a hard time in their current lives, rethink it from today’s guidance, and write a structured diary reflectively to describe their thoughts and insights.

All participants completed the Cyberostracism Experience Scale, *CD-RISC-25* Scale, and Prosocial Behavior Scale, and demographic information at day 0 (Time 1), day 7 (Time 2), day 14 (Time 3), and day 21 (Time 4) of the intervention. During the 21 days, participants in the intervention group received intervention tasks every day, whereas participants in the control group did not receive any tasks. All participants signed the informed consent form and received 200 or 50 CNY for their participation in intervention group or control group, respectively.

**Cyberostracism**. A self-developed 35-item Cyberostracism Experience Scale for local Chinese individuals was used (see Appendix A for details of the scale development process). Participants rated cyberostracism on a five-point Likert scale (1 = This situation has never happened to 5 = This situation always happens). The sample item was “In an online group chat, your speech is ignored or skipped by others”. The Cronbach’s α coefficients of the four waves of the scale in this study were 0.94, 0.96, 0.97, and 0.97.

**Psychological resilience**. As mentioned above, psychological resilience was assessed using 25 items adapted from Connor and Davidson [77]. This scale included five dimensions (the notion of personal competence, high standards, and tenacity, *NHT*; trust in one’s instincts, tolerance of negative affect, and strengthening effects of stress, *TT*; the positive acceptance of change, and secure relationships, *TPS*; related to control, *RC*; and spiritual influences, *SI*). The Cronbach’s α coefficients of the four waves of the scale in this study were 0.84, 0.88, 0.86, and 0.89, respectively.

**Prosocial behavior**. Twelve self-developed conditions about helping (see Appendix E) were used following Twenge et al. [67]. After reading each condition, we asked the participants two questions in turn: “Faced with this situation, are you willing to help?” (1 = very unwilling to 7 = very willing); “How much are you willing to help?” (this question varied depending on different helping conditions, with generally 1= offer very less help to 7= offer very much help). The twelve helping conditions were randomly distributed to the four-time points with three conditions each time.

At the end of the questionnaire, demographic information, such as gender and age, education, income, length of time surfing the net, daily hours spent online, and daily hours spent on social media, were collected.

### 3.2. Results

Before the intervention, the chi-squared test revealed that there were no significant gender differences between the two groups (*X*^2^(1) = 0.78, *p* = 0.379), and the *t* test revealed that there were no significant differences between the two groups in age (*t*(61) = −0.23, *p* = 0.817), education (*t*(61) = 0.21, *p* = 0.837), income (*t*(61) = −1.43, *p* = 0.158), length of surfing the net (*t*(61) = 0.70, *p* = 0.487), hours spent online daily (*t*(61) = 0.86, *p* = 0.394), hours spent on social media daily (*t*(61) = −0.36, *p* = 0.717), cyberostracism (*t*(61) = 0.12, *p* = 0.909), psychological resilience (*t*(61) = −1.17, *p* = 0.246), and prosocial behavior (*t*(61) = −1.01, *p* = 0.316).

Then, we examined the intervention effect. First, the results showed that in the intervention group, except for spiritual influences (*t*(31) = 1.58, *p* = 0.125), participants’ scores for psychological resilience (*t*(31) = 4.17, *p* < 0.001) and the other four dimensions (*t*(31) = 2.94, *p* = 0.006) at Time 4 were significantly higher than those at Time 1. For participants in the control group, there were no significant differences between Time 1 and Time 4 in psychological resilience (*t*(30) = 0.14, *p* = 0.891) or the other five dimensions (*t*(30) = 0.24, *p* = 0.117).

To determine the practice effect generated by the repeated measurement of psychological resilience on the results, the *T* test at Time 4 revealed that there were significant differences between the two groups in psychological resilience (*t*(61) = 2.57, *p* = 0.013), *TPS* (*t*(61) = 3.66, *p* = 0.001), *RC* (*t*(61) = 2.52, *p* = 0.014), and *SI* (*t*(61) = 2.15, *p* = 0.036). However, the differences between the two groups in *NHT* (*t*(61) = 1.55, *p* = 0.128) and *TT* (*t*(61) = 1.14, *p* = 0.260) were not significant. These results revealed that the intervention of psychological resilience still produced an effect on participants after eliminating the practice effect.

**Figure 2:**
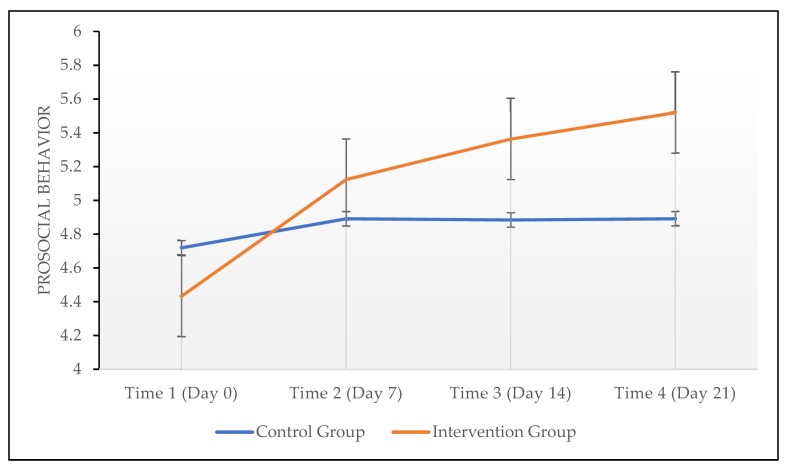
Effects of repeated measures on prosocial behavior. Error bars represent the 95% confidence interval.

Finally, we conducted repeated measures 2 (group: intervention, control) × 4 (time: days 0, 7, 14, and 21) mixed *ANCOVA* on prosocial behavior, with gender, age, education, income, length of surfing the net, hours spent online daily, and hours spent on social media daily as covariates. As seen in Figure 2, the results showed that the main effect of time was not significant (*F*(2, 94) = 0.52, *p* = 0.569, *p-η^2^* = 0.010), and the effect of the interaction between time and group was significant (*F*(2, 94) = 6.83, *p* = 0.003, *p-η^2^* = 0.112). Specifically, there were no significant differences among the four time points (*F*(1, 54) < 2.98, *p* = 0.090, *p-η^2^* = 0.052) in prosocial behavior in the control group. However, in the intervention group, there was a significant difference between Time 1 and Time 2 in prosocial behavior (*F*(1, 54) = 10.18, *p* = 0.002, *p-η^2^* = 0.159), but no significant differences between Time 2 and Time 3 (*F*(1, 54) = 2.02, *p* = 0.161, *p-η^2^* = 0.036), or between Time 3 and Time 4 (*F*(1, 54) = 0.62, *p* = 0.435, *p-η^2^* = 0.011) in prosocial behavior, indicating that the intervention effect mainly worked effectively between Times 1 and 2.

## 4. General Discussion

In conclusion, two studies were conducted in the current research to examine the relationship between cyberostracism and prosocial behaviors, as well as the role of psychological resilience in this relationship. Study 1 demonstrated that psychological resilience was helpful in mitigating the negative impact of cyberostracism on prosocial behaviors with experimental manipulation. Study 2 confirmed again that psychological resilience effectively alleviated the detrimental influence of cyberostracism on prosocial behavior by intervening in the psychological resilience of those who were cyberostracized with mental disorder and lacked resilience.

### 4.1. Implications

In Study 1, although we did not find the direct effect of cyberostracism on prosocial behaviors (i.e., the main effect), the alleviating effects of psychological resilience were supported. That is, the effect of cyberostracism on prosocial behaviors is not necessarily negative, and it mainly depends on individuals’ resilience. In other words, cyberostracism only causes fewer prosocial behaviors when among individuals low in psychological rather than those high in psychological resilience, indicating that psychological resilience was a significant protective factor that effectively alleviated the negative impact of cyberostracism on prosocial behaviors. The most important reason relates to the adaptability of psychological resilience [78]. The adaptability of psychological resilience is a benign adaptation of the individuals to life challenges through conscious adjustment and control, which helps individuals achieve effective regulation and adaptation to the external environment in dynamic changes. This positive psychological quality of resilience is the fundamental reason why psychological resilience played a protective role in the present study.

In addition, according to the temporal need–threat model of ostracism proposed by Williams [19,20], similar to instinctive response, the responses to ostracization in the reflexive stage are not affected by individual differences and situational factors; thus, there are no moderating variables in the immediate reflexive stage. However, in the present study, Study 1 showed that psychological resilience might have already played a moderating role in the reflexive stage. This finding may be explained by the definition of psychological resilience. Connor and Davidson [77] pointed out that psychological resilience is the ability to cope with negative life events such as stress, frustration, and trauma. When this kind of ability becomes a stable individual trait (i.e., schematization), it automatically helps individuals resist hazards instantaneously when they are confronted with cyberostracism [79]. This process can be described by Kumpfer’s [80] integrated model of resilience; that is, during the dynamic interaction between the individual and the environment, a high level of psychological resilience helps the individual transform the high-risk environment into a protective environment to facilitate resilience restructuring or active adaptation, such as selective awareness (focusing on the positive side of negative events) and cognitive restructuring (correcting irrational beliefs). Another explanation may lie in the fact that people with a high level of resilience tend to have more positive emotions [61,81,82], which aid in further expanding their instantaneous cognitive and behavioral ability [81,83] to function in the reflexive stage. Therefore, the present study may enrich the temporal need–threat model of ostracism by revealing that the cognitive mechanisms of resilience may already be involved in protective work in a timely manner during the reflexive stage in an online ostracism situation.

Rutter [84] emphasized the gene-environment interdependence of psychological resilience, and this gene-environment operates especially with respect to antisocial behavior. Resilience is a process and an interactive concept, rather than a fixed trait, that could be influenced by both personality dispositions and external systems [85]. Therefore, we can speculate that resilience could be improved by training, especially given that evidence has proven that a certain amount of adversity experience [86] and appropriate intervention [87] are conducive to the improvement of individuals’ psychological resilience. In Study 2, the online resilience intervention program we adopted had the characteristics of an individualized intervention under the same framework. Although participants read the same material in the first task, in the second structured diary writing task, participants were asked to analyze their problems reflectively based on their daily life experiences or events, and to propose solutions according to the rules we gave. To some extent, this intervention is a kind of cognitive pattern reconstruction training that gradually advances the psychological resilience of the participants, thus preventing a significant decrease in prosocial tendencies after ostracization online.

Therefore, the findings of Study 2 not only remind us that psychological resilience is beneficial to alleviate the negative impact of cyberostracism on prosocial behaviors, but also provide future researchers with an effective online self-help intervention program to help a larger population suffering from cyberostracism and poor resilience. Such a program could empower this population to increase their resilience through systematic web-based training without hurdles such as stigma, high time and financial commitments, or geographic restrictions, as it could be widely and readily accessed, and be capable of providing self-help in case of need [88,89,90,91]. This is the value of the present study.

### 4.2. Limitations and Future Directions

There are some limitations of the present study. First, cyberostracism might have some negative effects on psychological resilience, but we did not investigate such possible effects in this paper, which is a limitation and needs further study in the future.

Second, Ren et al. [92] found that individuals may also be more inclined to avoid social interactions and seek solitude to protect themselves from more social pain after ostracism, rather than to engage in the prosocial or antisocial tendencies mostly considered by researchers. Therefore, individuals’ desire to seek solitude after being ostracized online should be verified in the future.

Third, Study 1 showed that psychological resilience played a protective role in the reflection stage of ostracism, and we speculated that resilience might also have exerted a protective influence in the reflexive stage. Given the previous assertion that there were no moderating variables in the reflexive stage, this finding should be considered with caution, and we suggest that other researchers test this finding again. In addition, it is unclear whether resilience has the same effect in the resignation stage of ostracism. Consequently, future researchers should clarify whether the positive effects of psychological resilience exist in every stage of the temporal need–threat model of ostracism.

Finally, the internet-based self-help resilience intervention program developed in Study 2 is a novel positive attempt, and the results have already confirmed the effectiveness, efficacy, and feasibility of its implementation. By delivering this intervention electronically, many of the accessibility barriers were also addressed [88]. However, we still suggest that more research is needed to further provide evidence for its effectiveness, as it is still a rarely investigated area [91]. Admittedly, despite the resilience intervention in Study 2 being significantly effective, it is still doubtful how long the intervention effect lasts. Tagalidou et al. [93] pointed out that an important factor of successful intervention was the duration of intervention. Therefore, a continuous and stable intervention environment may be necessary for the long-term advancement of cognitive patterns.

## 5. Conclusions

The present study found that the temporal need–threat model of ostracism is also applicable to cyberostracism by showing the negative impact of cyberostracism on prosocial behavior. However, this effect was not consistent between groups of Chinese people with high and low resilience levels, as high resilience can mitigate the detrimental impact of cyberostracism on prosocial behavior, even from the reflexive stage of the temporal need–threat model. This alleviating effect was proven again in the online resilience intervention study. The web-based self-help resilience intervention program designed especially for people who are experiencing cyberostracism and lack resilience showed promising therapeutic effects, and is a valuable contribution to the present study. Hopefully, this web-based self-help resilience intervention program, which is free of a series of obstacles to delivery and access, could benefit more populations who are vulnerable to cyberostracism.

## Data Availability

The data presented in this study are available on request from the corresponding author.

## Appendix A

### Details of the Self-Developed 35-Item Cyberostracism Experience Scale Development Process

First, according to the frequency of internet usage, we invited 40 users (*female* = 23) to participate in structured interviews. We introduced the definition of cyberostracism to the interviewees, and then encouraged them to recall relevant experiences or talk about their opinions about this concept. Afterward, we introduced cyberostracism situations that had happened in the past, and then asked the interviewees whether they had experienced such situations recently. Additionally, they were encouraged to recall other cyberostracism situations that were not mentioned above.

After the interview, four Ph.D. students majoring in psychology sorted the interview notes and finally formed four main dimensions of cyberostracism, including *ignoring* (your existence is ignored unintentionally by others), *rejection* (others reject your clear signal to establish contact), *exclusion* (you are not accepted and excluded by the group), and *disparagement* (you are belittled, satirized, or slandered). The above process led to 120 items, including 32 items for ignoring, 32 items for rejection, 24 items for exclusion, and 32 items for disparagement.

Finally, we invited 400 participants to participate in the exploratory factor analysis. Thirty-nine failed the quality check questions, leading the final sample to 361 (*female* = 234, *M*_age_ = 28.36, *SD*_age_ = 5.91). The *KMO* test (*KMO* = 0.97) and Bartlett’s test of sphericity (*χ*^2^ = 11,058.87, *p* < 0.001) suggested that the correlation matrix was appropriate for factor analysis. Multiple criteria were used to determine the retention of factors, following Niu et al. [94]. The principal axis factoring method was used to extract the number of factors; the characteristic value was set to be greater than 1. The maximum number of iterations was set to 125. Oblique rotation was performed through the direct oblimin method; items with a load less than 0.3 or multiple cross-loadings were also removed. Finally, the result showed 35 items with a four-factor solution (ignoring, rejection, exclusion, disparagement), which explained 68.49% of the variance. Then, we conducted confirmatory factor analysis. We recruited 615 participants, 53 of whom failed the question check, resulting in a final sample of 562 participants (*female* = 340, *M*_age_ = 28.00, *SD*_age_ = 7.73). All models were tested on the 35 items. The results showed that the four-factor model fits the data better than alternative models. For instance, the four-factor model (*χ*^2^ = 1200.92, *df* = 554, *χ^2^/df* = 2.17, *ILI* = 0.95, *CFI* = 0.95, *RMSEA* = 0.05) yielded a better fit than the other models, which indicated that the constructs of cyberostracism had good discriminant validity. In the formal test, the Cronbach’s α coefficients for the scale was 0.96, for *ignoring* was 0.83, for *rejection* was 0.89, for *exclusion* was 0.89, and for *disparagement* was 0.95.

## Appendix B

### Appendix B.1. Manipulation Materials for Cyberostracism and Cyberacceptance

First, you need to complete mental imagery training, which is needed for the rest of the experiment. Please read the following story scenario carefully and try to imagine that you are the main character in the story. Please imagine that the story is happening in your real life as if you were there, and describe the specific scenes of the story in your mind: the more realistic, the better.

### Appendix B.2. Manipulation Materials for Cyberostracism

It is the end of the year, and you are planning to organize a New Year’s Eve event. After several months of careful planning, you create a business plan introducing the time, location, and process of the event, hoping that people would enthusiastically sign up to participate. You posted the invitation on each major online platform, but no one cares to ask about it, no one gives it a “like”, no one comments on it, no one retweets it, no one signs up……Later, you send the invitation to major online communities and talk about it, hoping that people will sign up and help retweet it, but your message is skipped in everyone’s chat, and no one pays attention to it. Later, you send the proposal to several close online communities, hoping that people will make comments and help you improve the proposal so that it could be used for the second round of releases, but your requests still do not get a response from people.

Since the venue, equipment, etc., have already been arranged, you still prepare yourself to hold the event on time. On the day, only people passing by the site come in to have a look, and few people participate, but it is good to see that it is not completely empty and that the sessions are still launched as planned. After finishing the work, you carefully organize the photos, videos, and other materials of the day, and create an event summary, which contains all the scene settings of the event day, the active atmosphere of the event, the perfect moments, and interesting highlights.

You post this summary of the event on all major online platforms, hoping that people will see it and sign up in droves the following year. But surprisingly, your post is met with numerous unfriendly, and even hurtful and malicious comments. People troll in the comment section with ridicule, sarcasm, and irony...... Some say your scene settings were terrible, some say your aesthetics were poor, some say your event design was uninteresting...... Even some people who participated in the event that day took photos, and post their personal updates, saying it was the worst event they had ever attended, and many people also like the updates or leave messages to express their approval. Then, you overhear some people in the online community discussing the event, and most of them are negative comments. You try to respond to them and explain, but get even more vitriol.

### Appendix B.3. Manipulation Materials for Cyberacceptance

It is the end of the year, and you are planning to organize a New Year’s Eve event. After several months of careful planning, you create a business plan introducing the time, location, and process of the event, hoping that people will enthusiastically sign up to participate. You post the invitation on each major online platform and receive an overwhelming response from people who like, praise, forward the invitation, and sign up. Later, you send the invitation to major online communities and talk about it in hopes that people will sign up and help forward it, and people see it and leave messages, saying they are willing to do so.

On the day of the event, the people who registered come as promised, the atmosphere is warm and cheerful, the event goes smoothly, and is a great success, and people have a pleasant evening. After finishing the work, you carefully organize the photos, videos, and other materials of the day, and create an event summary, which contains all the scene settings of the event day, the active atmosphere of the event, the perfect moments, and interesting highlights.

You post this summary of the event on all major online platforms, and once again, it garners wide acclaim. In the comment section, people enthusiastically discuss the happy details of the day’s activities; some say the scene settings were beautifully decorated, some say the desserts were delicious, and some say the games were full of surprises. There were also people who participated in the event that day who took photos and post personal updates, saying that it was the best event they had ever participated in, and many people like or leave comments on the updates to express their approval. You also see people in the online community talking enthusiastically about the event and sharing the joy of attending it with each other. You thank them for their recognition, and they thank you for your hard work.

## Appendix C

### Appendix C.1. Materials of the Five Self-Developed Prosocial Conditions about Donation

#### Appendix C.1.1. Condition 1

Xiaojun was found to have leukemia, and his father was an ordinary worker. Faced with the high cost of the transplant, the family could not afford it, and even the usual treatment expenses were already unaffordable.

#### Appendix C.1.2. Condition 2

Xiaoyang suffered from severe rheumatoid disease and was gradually unable to move. To give him treatment, family savings were spent, and the follow-up treatment costs left the family at a loss.

#### Appendix C.1.3. Condition 3

Juanzi is the mother of a 10-year-old seriously ill child in the city, operating a fruit stall to make ends meet. Not long ago, the child was sick for a long time in the hospital, and Juanzi had to give up the business to go to the hospital to take care of the child, with no source of income.

#### Appendix C.1.4. Condition 4

Xiaoming, an art major in a vocational high school for students with disabilities, was admitted to a special education vocational and technical college this year. However, his or her parents’ health is poor, and their income is meagre, so the living expenses and tuition fees for studying have become a difficult problem for the family.

#### Appendix C.1.5. Condition 5

A school in a remote area has poor basic conditions, with worn-out desks and chairs, rudimentary classrooms, and no computers and other equipment, but due to a lack of funds, it has not been improved, and the school is in great need of help from society to provide a better learning and living environment for the students.

## Appendix D

Materials of the online self-help resilience intervention program (website).

Intervention group:

Day 0: https://www.credamo.com/s/fIRJba. (accessed on 16 May 2021)

Day 1: https://www.credamo.com/s/6viIFn. (accessed on 17 May 2021)

Day 2: https://www.credamo.com/s/BnABFj. (accessed on 18 May 2021)

Day 3: https://www.credamo.com/s/uIfium. (accessed on 19 May 2021)

Day 4: https://www.credamo.com/s/VJ7jqy. (accessed on 20 May 2021)

Day 5: https://www.credamo.com/s/yiiQv2. (accessed on 21 May 2021)

Day 6: https://www.credamo.com/s/26bUzy. (accessed on 22 May 2021)

Day 7: https://www.credamo.com/s/JjmMR3. (accessed on 23 May 2021)

Day 8: https://www.credamo.com/s/R7b2y2. (accessed on 24 May 2021)

Day 9: https://www.credamo.com/s/yaqINz. (accessed on 25 May 2021)

Day 10: https://www.credamo.com/s/Efeiqm. (accessed on 26 May 2021)

Day 11: https://www.credamo.com/s/EBNRje. (accessed on 27 May 2021)

Day 12: https://www.credamo.com/s/UNvUva. (accessed on 28 May 2021)

Day 13: https://www.credamo.com/s/ziIJBb. (accessed on 29 May 2021)

Day 14: https://www.credamo.com/s/f63Ijy. (accessed on 30 May 2021)

Day 15: https://www.credamo.com/s/E3ABRz. (accessed on 31 May 2021)

Day 16: https://www.credamo.com/s/J3AJBf. (accessed on 1 June 2021)

Day 17: https://www.credamo.com/s/36jUNn. (accessed on 2 June 2021)

Day 18: https://www.credamo.com/s/FB7jUf. (accessed on 3 June 2021)

Day 19: https://www.credamo.com/s/fqYrQj. (accessed on 4 June 2021)

Day 20: https://www.credamo.com/s/aIVRNv. (accessed on 5 June 2021)

Day 21: https://www.credamo.com/s/Fvqa6j. (accessed on 6 June 2021)

Control group:

Day 0: https://www.credamo.com/s/jMBZbq. (accessed on 16 May 2021)

Day 7: https://www.credamo.com/s/mAveeq. (accessed on 23 May 2021)

Day 14: https://www.credamo.com/s/E3AbIb. (accessed on 30 May 2021)

Day 21: https://www.credamo.com/s/eaAvUj. (accessed on 6 June 2021)

## Appendix E

### Appendix E.1. Materials of the Twelve Self-Developed Prosocial Conditions about Helping

#### Appendix E.1.1. Condition 1

After you have completed a research task for a group of researchers and received your fee, the researchers ask you if you would like to help with some more experiments that take 5 min each, and you can choose not to do them or do as many as you want without affecting your current fee and without additional payment: “Would you like to help?” (1 = very unwilling to 7 = very willing); “How many more experiments would you like to help with?” (1 = 0, 2 = 1–2, 3 = 3–4, 4 = 5–6, 5 = 7–8, 6 = 9–10, 7 = more than 10).

#### Appendix E.1.2. Condition 2

A high school student is suffering from depression and is participating in a study on depression treatment. The study shows that if depressed people receive encouraging messages from others, it can improve their emotional state and help them recover: “Would you be willing to write a message of encouragement to this depressed high school student?”; “How long would you be willing to take to compile and write this message?” (1 = 3 min, 2 = 6 min, 3 = 9 min, 4 = 12 min, 5 = 15 min, 6 = 18 min, 7 = more than 18 min).

#### Appendix E.1.3. Condition 3

You pass by a medical volunteer recruitment event, which involves sending condolences to elderly people with no family at the local home for elderly people. They were happy to be visited in the past for approximately half a day each time: “Would you be willing to sign up for it?”; “How many times would you be willing to sign up for this?” (1 = 0 time, 2 = 1–2 times, 3 = 3–4 times, 4 = 5–6 times, 5 = 7–8 times, 6 = 9–10 times, 7 = more than 10 times).

#### Appendix E.1.4. Condition 4

A community is recruiting volunteers to take care of children in a local orphanage, including storytelling, games, etc. In the past, the children were very happy every time, looking forward to the next time there would be aunts and uncles to come; the activity is half a day long each time: “Would you like to sign up to participate?”; “How many times would you be willing to sign up?” (1 = 0 time, 2 = 1–2 times, 3 = 3–4 times, 4 = 5–6 times, 5 = 7–8 times, 6 = 9–10 times, 7 = more than 10 times).

#### Appendix E.1.5. Condition 5

On a crowded commuter public transport, you meet an elderly person who has no seat on board; if you have a seat and the elderly person happens to be standing next to you, “Would you be willing to give up your seat to him or her?”; “If there are many stops along the way and you encounter many situations where you need to give up your seat, how many times would you be willing to do so?” (1 = 0 times, 2 = 1–2 times, 3 = 3–4 times, 4 = 5–6 times, 5 = 7–8 times, 6 = 9–10 times, 7 = more than 10 times).

#### Appendix E.1.6. Condition 6

A rehabilitation center for deaf people has a group of hearing-impaired patients admitted, and is currently recruiting volunteer sign language teachers to train them, and you happen to be proficient in sign language: “Are you willing to sign up for volunteer activities?”; “How many times would you be willing to sign up?” (1 = 0 time, 2 = 1–2 times, 3 = 3–4 times, 4 = 5–6 times, 5 = 7–8 times, 6 = 9–10 times, 7 = more than 10 times).

#### Appendix E.1.7. Condition 7

You receive a drift bottle online describing the painful emotions of a bereaved person hoping to receive support and comfort from others: “Would you like to reply to this drift bottle?”; “How many words of comfort would you like to write for him or her?” (1 = less than 20 words, 2 = 20–40 words, 3 = 40–60 words, 4 = 60–80 words, 5 = 80–100 words, 6 = 100–120 words, 7 = more than 120 words).

#### Appendix E.1.8. Condition 8

During the Spring Festival, a group of freshmen tried online ticketing for the first time and had many difficulties, and found you to help, as you already have a lot of experience in ticketing: “Are you willing to help scramble for tickets?”; “How many students are you willing to help?” (1 = less than 2 students, 2 = 4 students, 3 = 6 students, 4 = 8 students, 5 = 10 students, 6 = 12 students, 7 = more than 12 students).

#### Appendix E.1.9. Condition 9

Near the final exam or year-end assessment, the next class of underclassmen or new colleagues asks you for information about your experience in passing the exam or assessment last year: “Would you be willing to offer your help?”; “What percent of your information would you be willing to share with them?” (1 = within 15%, 2 = 15–30%, 3 = 30–45%, 4 = 45–60%, 5 = 60–75%, 6 = 75–90%, 7 = more than 90%).

#### Appendix E.1.10. Condition 10

On the weekend, you were fishing at the lake, and there was a child around you who was also fishing. Toward the evening, you caught many fish, but the child did not catch any of them and was in a very depressed mood, and he looked at your bucket of fish with great envy: “Would you be willing to share your fish with him?”; “What percentage of your catch would you be willing to share?” (1 = within 15%, 2 = 15–30%, 3 = 30–45%, 4 = 45–60%, 5 = 60–75%, 6 = 75–90%, 7 = more than 90%).

#### Appendix E.1.11. Condition 11

Faced with an incident of an innocent person being the victim of cyber violence on the internet, you sympathize with the victim in the incident: “Will you support him or her and condemn the keyboard warriors in the comment section?”; “How many words would you write down to express your comfort to the victim and condemnation to the perpetrators?” (1 = within 20 words, 2 = 20–40 words, 3 = 40–60 words, 4 = 60–80 words, 5 = 80–100 words, 6 = 100–120 words, 7 = more than 120 words).

#### Appendix E.1.12. Condition 12

On the internet, you see a notice of a shortage of supplies in disaster areas, and want people to help forward it: “Would you be willing to help forward it?”; “On how many social platforms/chat groups are you willing to help forward it?” (1 = 0, 2 = 1–2, 3 = 3–4, 4 = 5–6, 5 = 7–8, 6 = 9–10, 7 = more than 10).
